# Padé approximant related to inequalities involving the constant *e* and a generalized Carleman-type inequality

**DOI:** 10.1186/s13660-017-1479-8

**Published:** 2017-09-04

**Authors:** Chao-Ping Chen, Hui-Jie Zhang

**Affiliations:** School of Mathematics and Informatics, Henan Polytechnic University, Jiaozuo City, Henan Province 454000 China

**Keywords:** 26D15, 41A60, Carleman’s inequality, weight coefficient, Padé approximant

## Abstract

Based on the Padé approximation method, in this paper we determine the coefficients $a_{j}$ and $b_{j}$ ($1\leq j \leq k$) such that

$$ \frac{1}{e} \biggl( 1+\frac{1}{x} \biggr) ^{x}= \frac{x^{k}+a_{1}x^{k-1}+ \cdots +a_{k}}{x^{k}+b_{1}x^{k-1}+\cdots +b_{k}}+O \biggl( \frac{1}{x ^{2k+1}} \biggr) , \quad x\to \infty , $$

where $k\geq 1$ is any given integer. Based on the obtained result, we establish new upper bounds for $( 1+1/x ) ^{x}$. As an application, we give a generalized Carleman-type inequality.

## Introduction

Let $a_{n} \geq 0$ for $n \in \mathbb{N}:=\{1, 2, \ldots \}$ and $0<\sum_{n=1}^{\infty }a_{n}<\infty $. Then

1.1 $$ \sum_{n=1}^{\infty }(a_{1}a_{2} \cdots a_{n})^{1/n}< e \sum_{n=1}^{ \infty }a_{n}. $$

The constant *e* is the best possible. The inequality (1.1) was presented in 1922 in [1] by Carleman and it is called Carleman’s inequality. Carleman discovered this inequality during his important work on quasi-analytical functions.

Carleman’s inequality (1.1) was generalized by Hardy [2] (see also [3, p.256]) as follows: If $a_{n} \geq 0$, $\lambda_{n}>0$, $\Lambda_{n}=\sum_{m=1}^{n}\lambda_{m}$ for $n \in \mathbb{N}$, and $0<\sum_{n=1}^{\infty }\lambda_{n}a_{n}< \infty $, then

1.2 $$ \sum_{n=1}^{\infty } \lambda_{n} \bigl(a_{1}^{\lambda_{1}}a_{2}^{\lambda_{2}} \cdots a_{n}^{\lambda_{n}} \bigr)^{1/ \Lambda_{n}}< e \sum _{n=1} ^{ \infty }\lambda_{n}a_{n}. $$

Note that inequality (1.2) is usually referred to as a Carleman-type inequality or weighted Carleman-type inequality. In [2], Hardy himself said that it was Pólya who pointed out this inequality to him.

In [4–20], some strengthened and generalized results of (1.1) and (1.2) have been given by estimating the weight coefficient $( 1+1/n ) ^{n}$. For example, Yang [17] proved that, for $n\in \mathbb{N}$,

1.3 $$ e \biggl( 1-\frac{1}{2(n+\frac{5}{6})} \biggr) < \biggl( 1+ \frac{1}{n} \biggr) ^{n}< e \biggl( 1-\frac{1}{2(n+1)} \biggr) , $$

and then used it to obtain the following strengthened Carleman inequality:

1.4 $$ \sum_{n=1}^{\infty }(a_{1}a_{2} \cdots a_{n})^{1/n}< e \sum_{n=1}^{ \infty } \biggl( 1-\frac{1}{2(n+1)} \biggr) a_{n}. $$

Xie and Zhong [15] proved that, for $x\geq 1$,

1.5 $$ e \biggl( 1-\frac{7}{14x+12} \biggr) < \biggl( 1+ \frac{1}{x} \biggr) ^{x}< e \biggl( 1-\frac{6}{12x+11} \biggr) , $$

and then used it to improve the Carleman-type inequality (1.2) as follows. If $0<\lambda_{n+1} \leq \lambda_{n}$, $\Lambda_{n}=\sum_{m=1}^{n}\lambda_{m}$, $a_{n} \geq 0$ for $n \in \mathbb{N}$, and $0<\sum_{n=1}^{\infty }\lambda_{n}a_{n}< \infty $, then

1.6 $$\begin{aligned} \sum_{n=1}^{\infty } \lambda_{n+1} \bigl(a_{1}^{\lambda_{1}}a_{2}^{ \lambda_{2}} \cdots a_{n}^{\lambda_{n}} \bigr)^{1/ \Lambda_{n}} < e\sum _{n=1} ^{\infty } \biggl( 1- \frac{6}{12(\frac{\Lambda_{n}}{\lambda_{n}})+11} \biggr) \lambda_{n}a _{n}. \end{aligned}$$

Taking $\lambda_{n} \equiv 1$ in (1.6) yields

1.7 $$\begin{aligned} \sum_{n=1}^{\infty }(a_{1}a_{2} \cdots a_{n})^{1/n} < e\sum_{n=1}^{ \infty } \biggl( 1-\frac{6}{12n+11} \biggr) a_{n}, \end{aligned}$$

which improves (1.4).

Recently, Mortici and Hu [14] proved that, for $x\geq 1$,

1.8 $$\begin{aligned} &\frac{x+\frac{5}{12}}{x+\frac{11}{12}}-\frac{5}{288x^{3}}+\frac{343}{8{,}640x^{4}} -\frac{2{,}621}{41{,}472x^{5}} \\ &\quad < \frac{1}{e} \biggl( 1+\frac{1}{x} \biggr)^{x} < \frac{x+\frac{5}{12}}{x+\frac{11}{12}}-\frac{5}{288x^{3}}+\frac{343}{8{,}640x ^{4}}-\frac{2{,}621}{41{,}472x^{5}}+ \frac{300{,}901}{3{,}483{,}648x^{6}}, \end{aligned}$$

and then they used it to establish the following improvement of Carleman’s inequality:

$$\begin{aligned} &\sum_{n=1}^{\infty }(a_{1}a_{2} \cdots a_{n})^{1/n} \\ &\quad < e \sum_{n=1}^{ \infty } \biggl( \frac{12n+5}{12n+11}-\frac{5}{288n^{3}}+\frac{343}{8{,}640n ^{4}}- \frac{2{,}621}{41{,}472n^{5}}+\frac{300{,}901}{3{,}483{,}648n^{6}} \biggr) a _{n}, \end{aligned}$$

which can be written as

1.9 $$\begin{aligned} \sum_{n=1}^{\infty }(a_{1}a_{2} \cdots a_{n})^{1/n}< e \sum_{n=1}^{ \infty } ( 1-\varepsilon_{n} ) a_{n}, \end{aligned}$$

where

1.10 $$\begin{aligned} \varepsilon_{n}=\frac{104{,}509{,}440n^{6}+3{,}628{,}800n^{4}-4{,}971{,}456n^{3}+5{,}603{,}472n ^{2}-5{,}945{,}040n-16{,}549{,}555}{17{,}418{,}240n^{6}(12n+11)}. \end{aligned}$$

For information as regards the history of Carleman-type inequalities, please refer to [21–24].

It follows from (1.8) that

1.11 $$ \frac{1}{e} \biggl( 1+\frac{1}{x} \biggr) ^{x}=\frac{x+\frac{5}{12}}{x+ \frac{11}{12}}+O \biggl( \frac{1}{x^{3}} \biggr) ,\quad x\to \infty . $$

Using the Padé approximation method, in Section 3 we derive (1.11) and the following approximation formula:

1.12 $$ \frac{1}{e} \biggl( 1+\frac{1}{x} \biggr) ^{x}=\frac{x^{2}+ \frac{87}{100}x+ \frac{37}{240}}{x^{2}+\frac{137}{100}x+ \frac{457}{1{,}200}}+O \biggl( \frac{1}{x ^{5}} \biggr) , \quad x\to \infty . $$

Equation (1.12) motivates us to present the following inequality:

1.13 $$ \biggl( 1+\frac{1}{n} \biggr) ^{n}< e \biggl( \frac{n^{2}+\frac{87}{100}n+ \frac{37}{240}}{n^{2}+\frac{137}{100}n+\frac{457}{1{,}200}} \biggr) =e \biggl( 1-\frac{8(75n+34)}{1{,}200n^{2}+1{,}644n+457} \biggr) , \quad n\in \mathbb{N}. $$

Following the same method used in the proof of Theorem 3.2, we can prove the inequality (1.13). We here omit it.

According to Pólya’s proof of (1.1) in [25],

1.14 $$ \sum_{n=1}^{\infty }(a_{1}a_{2} \cdots a_{n})^{1/n}\leq \sum_{n=1} ^{\infty } \biggl( 1+\frac{1}{n} \biggr) ^{n}a_{n}, $$

and then the following strengthened Carleman’s inequality is derived directly from (1.13):

1.15 $$ \sum_{n=1}^{\infty }(a_{1}a_{2} \cdots a_{n})^{1/n}< e \sum_{n=1}^{ \infty } \biggl( 1-\frac{8(75n+34)}{1{,}200n^{2}+1{,}644n+457} \biggr) a_{n}, $$

which improves (1.7).

Based on the Padé approximation method, we determine the coefficients $a_{j}$ and $b_{j}$ ($1\leq j\leq k$) such that

1.16 $$ \frac{1}{e} \biggl( 1+\frac{1}{x} \biggr) ^{x}=\frac{x^{k}+a_{1}x^{k-1}+ \cdots +a_{k}}{x^{k}+b_{1}x^{k-1}+\cdots +b_{k}}+O \biggl( \frac{1}{x ^{2k+1}} \biggr) ,\quad x\to \infty , $$

where $k\geq 1$ is any given integer. Based on the obtained result, we establish new upper bounds for $( 1+1/x ) ^{x}$. As an application, we give a generalization to the Carleman-type inequality.

The numerical values given have been calculated using the computer program MAPLE 13.

## A useful lemma

For later use, we introduce the following set of partitions of an integer $n \in \mathbb{N} =\mathbb{N}_{0} \setminus \{ 0 \} := \{1,\,2,\,3,\,\ldots \}$:

2.1 $$ \mathcal{A}_{n} := \bigl\{ ( k_{1}, k_{2}, \ldots , k_{n} ) \in \mathbb{N}_{0}^{n} : k_{1}+2k_{2}+ \cdots +nk_{n}=n \bigr\} . $$

In number theory, the partition function $p(n)$ represents the number of possible partitions of $n \in \mathbb{N}$ (*e.g.*, the number of distinct ways of representing *n* as a sum of natural numbers regardless of order). By convention, $p(0) = 1$ and $p(n) = 0$ if *n* is a negative integer. For more information on the partition function $p(n)$, please refer to [26] and the references therein. The first values of the partition function $p(n)$ are (starting with $p(0)=1$) (see [27]):

$$ 1, \,1,\,2, \,3,\,5,\,7,\,11,\,15,\,22,\,30, \,42,\,\ldots . $$

It is easy to see that the cardinality of the set $\mathcal{A}_{n}$ is equal to the partition function $p(n)$. Now we are ready to present a formula which determines the coefficients $a_{j}$ in (2.2) with the help of the partition function given by the following lemma.

### Lemma 2.1

[28]

*The following approximation formula holds true*:

2.2 $$ \biggl( 1+\frac{1}{x} \biggr) ^{x}=e \sum _{j=0}^{\infty } \frac{c _{j}}{x ^{j}} \quad \textit{as}\ x\to \infty , $$

*where the coefficients* $c_{j}$ $(j \in \mathbb{N})$ *are given by*

2.3 $$ \begin{aligned}& c_{0}=1 \quad \textit{and}\quad c_{j}=(-1)^{j} \sum_{ ( k_{1}, k_{2}, \ldots , k_{j} ) \in \mathcal{A} _{j}} \frac{1}{k_{1}!k_{2}!\cdots k_{j}!} \biggl( \frac{1}{2} \biggr) ^{k_{1}} \biggl( \frac{1}{3} \biggr) ^{k_{2}}\cdots \biggl( \frac{1}{j+1} \biggr) ^{k_{j}},\end{aligned}$$

*where the* $\mathcal{A}_{j}$ $(\textit{for}\ j \in \mathbb{N})$ *are given in* (2.1).

## Padé approximant related to asymptotics for the constant *e*

For later use, we introduce the Padé approximant (see [29–34]). Let *f* be a formal power series

3.1 $$\begin{aligned} f(t)=c_{0}+c_{1}t+c_{2}t^{2}+ \cdots . \end{aligned}$$

The Padé approximation of order $(p, q)$ of the function *f* is the rational function, denoted by

3.2 $$\begin{aligned}{} [p/q]_{f}(t)=\frac{\sum_{j=0}^{p}a_{j}t^{j}}{1+\sum_{j=1}^{q}b_{j}t ^{j}}, \end{aligned}$$

where $p\geq 0$ and $q\geq 1$ are two given integers, the coefficients $a_{j}$ and $b_{j}$ are given by (see [29–31, 33, 34])

3.3 $$\begin{aligned} \textstyle\begin{cases} a_{0}=c_{0}, \\ a_{1}=c_{0}b_{1}+c_{1}, \\ a_{2}=c_{0}b_{2}+c_{1}b_{1}+c_{2}, \\ \vdots \\ a_{p} = c_{0}b_{p}+\cdots + c_{p-1}b_{1} + c_{p}, \\ 0 = c_{p+1} + c_{p}b_{1} + \cdots + c_{p-q+1}b_{q}, \\ \vdots & \\ 0 = c_{p+q} + c_{p+q-1}b_{1} + \cdots + c_{p}b_{q}, \end{cases}\displaystyle \end{aligned}$$

and the following holds:

3.4 $$\begin{aligned}{} [p/q]_{f}(t)- f (t) = O\bigl(t^{p+q+1} \bigr). \end{aligned}$$

Thus, the first $p + q + 1$ coefficients of the series expansion of $[p/q]_{f}$ are identical to those of *f*. Moreover, we have (see [32])

3.5 $$ \begin{aligned} &[p/q]_{f}(t)= \frac{ \left \vert \begin{matrix}{} t^{q}f_{p-q}(t) & t^{q-1}f_{p-q+1}(t) &\cdots &f_{p}(t) \\ c_{p-q+1} & c_{p-q+2} &\cdots &c_{p+1} \\ \vdots &\vdots &\ddots &\vdots \\ c_{p} & c_{p+1} &\cdots &c_{p+q} \end{matrix} \right \vert } { \left \vert \begin{matrix}{} t^{q} & t^{q-1} &\cdots &1 \\ c_{p-q+1} & c_{p-q+2} &\cdots &c_{p+1} \\ \vdots &\vdots &\ddots &\vdots \\ c_{p} & c_{p+1} &\cdots &c_{p+q} \end{matrix} \right \vert } , \end{aligned} $$

with $f_{n}(x) = c_{0}+ c_{1}x+ \cdots + c_{n}x^{n}$, the *n*th partial sum of the series *f* ($f_{n}$ is identically zero for $n < 0$).

Let

3.6 $$\begin{aligned} f(x)=\frac{1}{e} \biggl( 1+\frac{1}{x} \biggr) ^{x}. \end{aligned}$$

It follows from (2.2) that, as $x\to \infty $,

3.7 $$\begin{aligned} f(x) &=\sum_{j=0}^{\infty } \frac{c_{j}}{x^{j}}=1-\frac{1}{2x}+\frac{11}{24x ^{2}}-\frac{7}{16x^{3}}+ \frac{2{,}447}{5{,}760x^{4}}-\frac{959}{2{,}304x^{5}}+\frac{238{,}043}{580{,}608x ^{6}}-\cdots , \end{aligned}$$

with the coefficients $c_{j}$ given by (2.3). In what follows, the function *f* is given in (3.6).

We now give a derivation of equation (1.11). To this end, we consider

$$\begin{aligned}{} [1/1]_{f}(x)=\frac{\sum_{j=0}^{1}a_{j}x^{-j}}{1+\sum_{j=1}^{1}b_{j}x ^{-j}}. \end{aligned}$$

Noting that

3.8 $$\begin{aligned} c_{0}=1, \qquad c_{1}=-\frac{1}{2},\qquad c_{2}=\frac{11}{24}, \qquad c _{3}=- \frac{7}{16}, \qquad c_{4}=\frac{2{,}447}{5{,}760} \end{aligned}$$

holds, we have, by (3.3),

$$\begin{aligned} \textstyle\begin{cases} a_{0}=1, \\a_{1}=b_{1}-\frac{1}{2}, \\0 =\frac{11}{24}- \frac{1}{2}b_{1}, \end{cases}\displaystyle \end{aligned}$$

that is,

$$\begin{aligned} a_{0}=1, \qquad a_{1}=\frac{5}{12},\qquad b_{1}=\frac{11}{12}. \end{aligned}$$

We thus obtain

3.9 $$ [1/1]_{f}(x)= \frac{1+\frac{5}{12x}}{1+\frac{11}{12x}}=\frac{x+ \frac{5}{12}}{x+\frac{11}{12}}, $$

and we have, by (3.4),

3.10 $$ \frac{1}{e} \biggl( 1+\frac{1}{x} \biggr) ^{x}-\frac{x+\frac{5}{12}}{x+ \frac{11}{12}}= O \biggl( \frac{1}{x^{3}} \biggr) , \quad x\to \infty . $$

We now give a derivation of equation (1.12). To this end, we consider

$$\begin{aligned}{} [2/2]_{f}(x)=\frac{\sum_{j=0}^{2}a_{j}x^{-j}}{1+\sum_{j=1}^{2}b_{j}x ^{-j}}. \end{aligned}$$

Noting that (3.8) holds, we have, by (3.3),

$$\begin{aligned} \textstyle\begin{cases} a_{0}=1, \\a_{1}=b_{1}-\frac{1}{2}, \\a_{2} =b_{2}-\frac{1}{2}b_{1}+ \frac{11}{24} , \\0 = - \frac{7}{16}+\frac{11}{24}b_{1}-\frac{1}{2}b_{2} , \\0 = \frac{2{,}447}{5{,}760}-\frac{7}{16}b_{1}+\frac{11}{24}b_{2}, \end{cases}\displaystyle \end{aligned}$$

that is,

$$\begin{aligned} a_{0}=1,\qquad a_{1}=\frac{87}{100},\qquad a_{2} = \frac{37}{240}, \qquad b_{1}=\frac{137}{100},\qquad b_{2}=\frac{457}{1{,}200}. \end{aligned}$$

We thus obtain

3.11 $$ [2/2]_{f}(x)= \frac{1+\frac{87}{100x}+\frac{37}{240x^{2}}}{1+ \frac{137}{100x}+\frac{457}{1{,}200x^{2}}}=\frac{x^{2}+\frac{87}{100}x+ \frac{37}{240}}{x^{2}+\frac{137}{100}x+\frac{457}{1{,}200}} $$

and we have, by (3.4),

3.12 $$ \frac{1}{e} \biggl( 1+\frac{1}{x} \biggr) ^{x}-\frac{x^{2}+ \frac{87}{100}x+ \frac{37}{240}}{x^{2}+\frac{137}{100}x+ \frac{457}{1{,}200}}= O \biggl( \frac{1}{x ^{5}} \biggr) , \quad x\to \infty . $$

Using the Padé approximation method and the expansion (3.7), we now present a general result given by Theorem 3.1. As a consequence, we obtain (1.16).

### Theorem 3.1

*The Padé approximation of order* $(p, q)$ *of the asymptotic formula of the function* $f(x)=\frac{1}{e} ( 1+\frac{1}{x} ) ^{x}$ (*at the point* $x=\infty $) *is the following rational function*:

3.13 $$\begin{aligned}{} [p/q]_{f}(x)=\frac{1+\sum_{j=1}^{p}a_{j}x^{-j}}{1+\sum_{j=1}^{q}b_{j}x ^{-j}}=x^{q-p} \biggl( \frac{x^{p}+a_{1}x^{p-1}+\cdots +a_{p}}{x^{q}+b _{1}x^{q-1}+\cdots +b_{q}} \biggr) , \end{aligned}$$

*where* $p\geq 1$ *and* $q\geq 1$ *are two given integers*, *the coefficients* $a_{j}$ *and* $b_{j}$ *are given by*

3.14 $$\begin{aligned} \textstyle\begin{cases} a_{1}=b_{1}+c_{1}, \\ a_{2}=b_{2}+c_{1}b_{1}+c_{2}, \\ \vdots \\ a_{p} = b_{p}+\cdots + c_{p-1}b_{1} + c_{p}, \\ 0 = c_{p+1} + c_{p}b_{1} + \cdots + c_{p-q+1}b_{q}, \\ \vdots & \\ 0 = c_{p+q} + c_{p+q-1}b_{1} + \cdots + c_{p}b_{q}, \end{cases}\displaystyle \end{aligned}$$

$c_{j}$ *is given in* (2.3), *and the following holds*:

3.15 $$\begin{aligned} f (x) -[p/q]_{f}(x) = O \biggl( \frac{1}{x^{p+q+1}} \biggr) ,\quad x\to \infty . \end{aligned}$$

*Moreover*, *we have*

3.16 $$ \begin{aligned} &[p/q]_{f}(x)= \frac{ \left \vert \begin{matrix}{} \frac{1}{x^{q}}f_{p-q}(x) & \frac{1}{x^{q-1}}f_{p-q+1}(x) &\cdots &f _{p}(x) \\c_{p-q+1} & c_{p-q+2} &\cdots &c_{p+1} \\ \vdots &\vdots &\ddots &\vdots \\ c_{p} & c_{p+1} &\cdots &c_{p+q} \end{matrix} \right \vert } { \left \vert \begin{matrix}{} \frac{1}{x^{q}} & \frac{1}{x^{q-1}} &\cdots &1 \\c_{p-q+1} & c_{p-q+2} &\cdots &c_{p+1} \\ \vdots &\vdots &\ddots &\vdots \\ c_{p} & c_{p+1} &\cdots &c_{p+q} \end{matrix} \right \vert } , \end{aligned} $$

*with* $f_{n}(x)=\sum_{j=0}^{n}\frac{c_{j}}{x^{j}}$, *the* *nth partial sum of the asymptotic series* (3.7).

### Remark 3.1

Using (3.16), we can also derive (3.9) and (3.11). Indeed, we have

$$ \begin{aligned}{} [1/1]_{f}(x) &=\frac{ \left \vert \begin{matrix}{} \frac{1}{x}f_{0}(x) &f_{1}(x) \\c_{1} &c_{2} \\\end{matrix} \right \vert } { \left \vert \begin{matrix}{} \frac{1}{x} &1 \\c_{1} &c_{2} \\\end{matrix} \right \vert } = \frac{ \left \vert \begin{matrix}{} \frac{1}{x} &1-\frac{1}{2x} \\-\frac{1}{2} &\frac{11}{24} \\\end{matrix} \right \vert } { \left \vert \begin{matrix}{} \frac{1}{x} &1 \\-\frac{1}{2} &\frac{11}{24} \\\end{matrix} \right \vert } \\ & =\frac{x+\frac{5}{12}}{x+\frac{11}{12}} \end{aligned} $$

and

$$ \begin{aligned}{} [2/2]_{f}(x) &=\frac{ \left \vert \begin{matrix}{} \frac{1}{x^{2}}f_{0}(x) & \frac{1}{x}f_{1}(x) &f_{2}(x) \\c_{1} &c_{2} &c_{3} \\c_{2} &c_{3} &c_{4} \\\end{matrix} \right \vert } { \left \vert \begin{matrix}{} \frac{1}{x^{2}} &\frac{1}{x} &1 \\c_{1} &c_{2} &c_{3} \\c_{2} &c_{3} &c_{4} \\\end{matrix} \right \vert } = \frac{ \left \vert \begin{matrix}{} \frac{1}{x^{2}} & \frac{1}{x} ( 1-\frac{1}{2x} ) &1- \frac{1}{2x}+\frac{11}{24x^{2}} \\-\frac{1}{2} &\frac{11}{24} &-\frac{7}{16} \\\frac{11}{24} &-\frac{7}{16} &\frac{2{,}447}{5{,}760} \\\end{matrix} \right \vert } { \left \vert \begin{matrix}{} \frac{1}{x^{2}} &\frac{1}{x} &1 \\-\frac{1}{2} &\frac{11}{24} &-\frac{7}{16} \\\frac{11}{24} &-\frac{7}{16} &\frac{2{,}447}{5{,}760} \\\end{matrix} \right \vert } \\ &=\frac{x^{2}+\frac{87}{100}x+\frac{37}{240}}{x^{2}+\frac{137}{100}x+ \frac{457}{1{,}200}}. \end{aligned} $$

### Remark 3.2

Setting $(p, q)=(k, k)$ in (3.15), we obtain (1.16).

Setting

$$ (p, q)=(3, 3) \quad \text{and}\quad (p, q)=(4, 4), $$

respectively, we obtain by Theorem 3.1, as $x\to \infty $,

3.17 $$ \frac{1}{e} \biggl( 1+\frac{1}{x} \biggr) ^{x}=\frac{x^{3}+ \frac{162{,}713}{121{,}212}x^{2}+\frac{13{,}927}{26{,}936}x+\frac{41{,}501}{786{,}240}}{x ^{3}+\frac{223{,}319}{121{,}212}x^{2}+\frac{237{,}551}{242{,}424}x+ \frac{3{,}950{,}767}{29{,}090{,}880}}+O \biggl( \frac{1}{x^{7}} \biggr) $$

and

3.18 $$\begin{aligned} \frac{1}{e} \biggl( 1+\frac{1}{x} \biggr) ^{x}={}&\frac{x^{4}+ \frac{1{,}157{,}406{,}727}{634{,}301{,}284}x^{3}+\frac{8{,}452{,}872{,}239}{7{,}611{,}615{,}408}x^{2}+ \frac{81{,}587{,}251{,}465}{319{,}687{,}847{,}136}x+\frac{15{,}842{,}677}{924{,}376{,}320}}{x^{4}+ \frac{1{,}474{,}557{,}369}{634{,}301{,}284}x^{3}+\frac{13{,}811{,}559{,}391}{7{,}611{,}615{,}408}x^{2}+ \frac{170{,}870{,}679{,}559}{319{,}687{,}847{,}136}x+ \frac{1{,}724{,}393{,}461{,}793}{38{,}362{,}541{,}656{,}320}} \\ & {}+O \biggl( \frac{1}{x^{9}} \biggr) . \end{aligned}$$

Equations (3.17) and (3.18) motivate us to establish the following theorem.

### Theorem 3.2

*For* $x>0$,

3.19 $$\begin{aligned} \biggl( 1+\frac{1}{x} \biggr) ^{x} &< e \biggl( \frac{x^{3}+ \frac{162{,}713}{121{,}212}x^{2}+\frac{13{,}927}{26{,}936}x+\frac{41{,}501}{786{,}240}}{x ^{3}+\frac{223{,}319}{121{,}212}x^{2}+\frac{237{,}551}{242{,}424}x+ \frac{3{,}950{,}767}{29{,}090{,}880}} \biggr) \end{aligned}$$

*and*

3.20 $$\begin{aligned} &\biggl( 1+\frac{1}{x} \biggr) ^{x} \\ &\quad < e \biggl( \frac{x^{4}+ \frac{1{,}157{,}406{,}727}{634{,}301{,}284}x^{3}+\frac{8{,}452{,}872{,}239}{7{,}611{,}615{,}408}x^{2}+ \frac{81{,}587{,}251{,}465}{319{,}687{,}847{,}136}x+\frac{15{,}842{,}677}{924{,}376{,}320}}{x^{4}+ \frac{1{,}474{,}557{,}369}{634{,}301{,}284}x^{3}+\frac{13{,}811{,}559{,}391}{7{,}611{,}615{,}408}x^{2}+ \frac{170{,}870{,}679{,}559}{319{,}687{,}847{,}136}x+ \frac{1{,}724{,}393{,}461{,}793}{38{,}362{,}541{,}656{,}320}} \biggr) . \end{aligned}$$

### Proof

We only prove the inequality (3.20). The proof of (3.19) is analogous. In order to prove (3.20), it suffices to show that

$$\begin{aligned} F(x)< 0 \quad \text{for}\ x>0, \end{aligned}$$

where

$$\begin{aligned} F(x)={}&x\ln \biggl( 1+\frac{1}{x} \biggr) -1 \\ &{}-\ln \biggl( \frac{x^{4}+ \frac{1{,}157{,}406{,}727}{634{,}301{,}284}x^{3}+\frac{8{,}452{,}872{,}239}{7{,}611{,}615{,}408}x^{2}+ \frac{81{,}587{,}251{,}465}{319{,}687{,}847{,}136}x+\frac{15{,}842{,}677}{924{,}376{,}320}}{x^{4}+ \frac{1{,}474{,}557{,}369}{634{,}301{,}284}x^{3}+\frac{13{,}811{,}559{,}391}{7{,}611{,}615{,}408}x^{2}+ \frac{170{,}870{,}679{,}559}{319{,}687{,}847{,}136}x+ \frac{1{,}724{,}393{,}461{,}793}{38{,}362{,}541{,}656{,}320}} \biggr) . \end{aligned}$$

Differentiation yields

$$\begin{aligned} F'(x)=\ln \biggl( 1+\frac{1}{x} \biggr) -\frac{P_{8}(x)}{P_{9}(x)}, \end{aligned}$$

where

$$\begin{aligned} P_{8}(x) ={}&4{,}534{,}960{,}145{,}139{,}175{,}220{,}907{,}601+89{,}156{,}435{,}404{,}854{,}709{,}617{,}164{,}400x \\ & +753{,}611{,}422{,}427{,}554{,}143{,}580{,}166{,}880x^{2} \\ & +3{,}400{,}732{,}641{,}706{,}885{,}239{,}015{,}784{,}320x^{3} \\ & +8{,}959{,}898{,}009{,}119{,}992{,}740{,}647{,}591{,}680x^{4} \\ & +14{,}212{,}846{,}466{,}921{,}911{,}377{,}490{,}790{,}400x^{5} \\ & +13{,}355{,}464{,}865{,}044{,}929{,}241{,}744{,}281{,}600x^{6} \\ & +6{,}842{,}437{,}276{,}900{,}714{,}847{,}214{,}796{,}800x^{7} \\ & +1{,}471{,}684{,}602{,}332{,}887{,}248{,}995{,}942{,}400x^{8} \end{aligned}$$

and

$$\begin{aligned} P_{9}(x) ={}&\bigl(38{,}362{,}541{,}656{,}320x^{4}+69{,}999{,}958{,}848{,}960x^{3}+42{,}602{,}476{,}084{,}560x ^{2} \\ &{}+9{,}790{,}470{,}175{,}800x +657{,}486{,}938{,}177\bigr) \bigl(38{,}362{,}541{,}656{,}320x^{4} \\ &{}+89{,}181{,}229{,}677{,}120x^{3}+69{,}610{,}259{,}330{,}640x ^{2} +20{,}504{,}481{,}547{,}080x \\ &{}+1{,}724{,}393{,}461{,}793\bigr) (x+1). \end{aligned}$$

Differentiating $F'(x)$, we find

$$\begin{aligned} F''(x)=-\frac{Q_{8}(x)}{Q_{19}(x)}, \end{aligned}$$

where

$$\begin{aligned} Q_{8}(x) ={}&1{,}285{,}425{,}745{,}031{,}439{,}744{,}924{,}351{,}944{,}181{,}267{,}498{,}830{,}297{,}392{,}321 \\ & +28{,}378{,}097{,}964{,}665{,}213{,}870{,}448{,}253{,}775{,}917{,}974{,}735{,}833{,}555{,}915{,}520x \\ & +247{,}639{,}239{,}538{,}550{,}650{,}618{,}428{,}925{,}475{,}351{,}177{,}418{,}903{,}828{,}519{,}360x^{2} \\ & +1{,}131{,}116{,}309{,}072{,}948{,}249{,}686{,}419{,}776{,}599{,}013{,}563{,}965{,}352{,}036{,}853{,}760x^{3} \\ & +2{,}998{,}129{,}273{,}934{,}033{,}621{,}834{,}452{,}343{,}529{,}577{,}599{,}070{,}175{,}646{,}117{,}120x^{4} \\ & +4{,}775{,}194{,}702{,}079{,}256{,}668{,}486{,}950{,}292{,}217{,}012{,}539{,}098{,}845{,}384{,}867{,}840x^{5} \\ & +4{,}503{,}188{,}365{,}939{,}207{,}771{,}317{,}966{,}173{,}833{,}346{,}921{,}724{,}385{,}791{,}590{,}400x^{6} \\ & +2{,}315{,}562{,}242{,}935{,}704{,}170{,}341{,}114{,}308{,}201{,}588{,}127{,}064{,}283{,}807{,}744{,}000x^{7} \\ & +500{,}009{,}489{,}498{,}922{,}911{,}594{,}629{,}442{,}997{,}057{,}334{,}195{,}586{,}408{,}448{,}000x^{8} \end{aligned}$$

and

$$\begin{aligned} Q_{19}(x) ={}&x\bigl(38{,}362{,}541{,}656{,}320x^{4}+69{,}999{,}958{,}848{,}960x^{3}+42{,}602{,}476{,}084{,}560x ^{2} \\ &+9{,}790{,}470{,}175{,}800x +657{,}486{,}938{,}177\bigr)^{2}\bigl(38{,}362{,}541{,}656{,}320x^{4} \\ &+89{,}181{,}229{,}677{,}120x^{3}+69{,}610{,}259{,}330{,}640x ^{2} +20{,}504{,}481{,}547{,}080x \\ &+1{,}724{,}393{,}461{,}793\bigr)^{2}(x+1)^{2}. \end{aligned}$$

Hence, $F''(x)<0$ for $x>0$, and we have

$$\begin{aligned} F'(x)>\lim_{t\to \infty }F'(t)=0 \quad \Longrightarrow \quad F(x)< \lim_{t\to \infty }F(t)=0 \quad \text{for}\ x>0. \end{aligned}$$

The proof is complete. □

The inequality (3.20) can be written as

3.21 $$\begin{aligned} \biggl( 1+\frac{1}{x} \biggr) ^{x}< e \bigl(1- \mathcal{E}(x) \bigr),\quad x>0, \end{aligned}$$

where

3.22 $$\begin{aligned} \mathcal{E}(x)={}& 48\bigl(399{,}609{,}808{,}920x^{3}+562{,}662{,}150{,}960x^{2} \\ &{}+223{,}208{,}570{,}235x+22{,}227{,}219{,}242\bigr) /\bigl(38{,}362{,}541{,}656{,}320x^{4} \\ &{}+89{,}181{,}229{,}677{,}120x^{3}+69{,}610{,}259{,}330{,}640x^{2}+20{,}504{,}481{,}547{,}080x \\ &{}+1{,}724{,}393{,}461{,}793\bigr). \end{aligned}$$

## A generalized Carleman-type inequality

### Theorem 4.1

*Let* $0<\lambda_{n+1} \leq \lambda_{n}$, $\Lambda_{n}=\sum_{m=1}^{n} \lambda_{m}$ $(\Lambda_{n}\geq 1)$, $a_{n} \geq 0$ $(n \in \mathbb{N})$ *and* $0<\sum_{n=1}^{\infty }\lambda_{n}a_{n}<\infty $. *Then*, *for* $0< p \leq 1$,

4.1 $$\begin{aligned} & \sum_{n=1}^{\infty } \lambda_{n+1}\bigl(a_{1}^{\lambda_{1}}a_{2}^{\lambda_{2}} \cdots a_{n}^{\lambda_{n}}\bigr)^{1/ \Lambda_{n}} \\ &\quad{} < \frac{e^{p}}{p} \sum_{n=1}^{\infty } \biggl( 1-\mathcal{E} \biggl( \frac{\Lambda_{n}}{ \lambda _{n}} \biggr) \biggr) ^{p} \lambda_{n}a_{n}^{p} \Lambda_{n}^{p-1} \Biggl( \sum_{k=1}^{n} \lambda_{k}(c_{k}a_{k})^{p} \Biggr) ^{(1-p)/p}, \end{aligned}$$

*where* $\mathcal{E}(x)$ *is given in* (3.22) *and*

$$ c_{n}^{\lambda_{n}}=\frac{(\Lambda_{n+1})^{\Lambda_{n}}}{(\Lambda_{n})^{ \Lambda_{n-1}}}. $$

### Proof

The inequality

4.2 $$\begin{aligned} & \sum_{n=1}^{\infty } \lambda_{n+1}\bigl(a_{1}^{\lambda_{1}}a_{2}^{\lambda_{2}} \cdots a_{n}^{\lambda_{n}}\bigr)^{1/ \Lambda_{n}} \\ &\quad \leq \frac{1}{p}\sum_{m=1}^{\infty } \biggl( 1+\frac{1}{\Lambda_{m}/ \lambda_{m}} \biggr) ^{p \Lambda_{m} / \lambda_{m}} \lambda_{m}a_{m} ^{p}\Lambda_{m}^{p-1} \Biggl( \sum _{k=1}^{m}\lambda_{k}(c_{k}a_{k})^{p} \Biggr) ^{(1-p)/p} \end{aligned}$$

has been proved in Theorem 2.2 of [9] (see also [11, p.96]). From the above inequality and (3.20), we obtain (4.1). The proof is complete. □

### Remark 4.1

In Theorem 2.2 of [9], $c_{k}^{\lambda_{n}}=\frac{( \Lambda_{n+1})^{\Lambda_{n}}}{(\Lambda_{n})^{\Lambda_{n-1}}}$ should be $c_{n}^{\lambda_{n}}=\frac{(\Lambda_{n+1})^{\Lambda_{n}}}{(\Lambda_{n})^{ \Lambda_{n-1}}}$; see [9, p.44, line 3]. Likewise, $c_{s}^{\lambda_{n}}=\frac{(\Lambda_{n+1})^{\Lambda_{n}}}{(\Lambda_{n})^{ \Lambda_{n-1}}}$ in Theorem 3.1 of [11] should be $c_{n}^{\lambda_{n}}=\frac{(\Lambda_{n+1})^{\Lambda_{n}}}{(\Lambda_{n})^{ \Lambda_{n-1}}}$; see [11, p.96, equation (9)].

### Remark 4.2

Taking $p=1$ in (4.1) yields

4.3 $$\begin{aligned} \sum_{n=1}^{\infty } \lambda_{n+1}\bigl(a_{1}^{\lambda_{1}}a_{2}^{\lambda_{2}} \cdots a_{n}^{\lambda_{n}}\bigr)^{1/ \Lambda_{n}} < e\sum _{n=1}^{ \infty } \biggl( 1-\mathcal{E} \biggl( \frac{\Lambda_{n}}{\lambda_{n}} \biggr) \biggr) \lambda_{n}a_{n}, \end{aligned}$$

which improves (1.6). Taking $\lambda_{n} \equiv 1$ in (4.3) yields

4.4 $$\begin{aligned} \sum_{n=1}^{\infty }(a_{1}a_{2} \cdots a_{n})^{1/n} < e\sum_{n=1}^{ \infty } \bigl(1-\mathcal{E}(n) \bigr)a_{n}, \end{aligned}$$

which improves (1.9).
